# Gd-EOB-DTPA-MRCP to localize bile leakage after liver trauma and surgery: impact on treatment and outcome

**DOI:** 10.1007/s00330-023-09608-x

**Published:** 2023-04-13

**Authors:** Adrian A. Marth, Timo A. Auer, Thula C. Walter-Rittel, Nora Nevermann, Felix Krenzien, Moritz Schmelzle, Tobias Müller, Johannes Kolck, Gero Wieners, Dominik Geisel, Bernhard Gebauer, Bernd Hamm, Wenzel Schöning, Uli Fehrenbach

**Affiliations:** 1 Department of Radiology, Charité - Universitätsmedizin Berlin, Augustenburger Platz 1, 13353 Berlin, Germany; 2 Berlin Institute of Health at Charité - Universitätsmedizin Berlin, Berlin, Germany; 3 Department of Surgery | CCM | CVK, Charité - Universitätsmedizin Berlin, Berlin, Germany; 4 Department of General, Visceral and Transplant Surgery, Medizinische Hochschule Hannover, Hannover, Germany; 5 Department of Hepatology and Gastroenterology, Charité - Universitätsmedizin Berlin, Berlin, Germany

**Keywords:** Biliary tract, Magnetic resonance imaging, Diagnostic imaging

## Abstract

**Objectives:**

Bile leakage (BL) is a challenging complication after hepatobiliary surgery and liver trauma. Gadolinium ethoxybenzyl (Gd-EOB-DTPA)–enhanced magnetic resonance cholangiopancreatography (MRCP) is used to diagnose BL non-invasively. We assessed the value of Gd-EOB-DTPA-MRCP in the detection of postoperative and post-traumatic BL hypothesizing that exact identification of the leakage site is pivotal for treatment planning and outcome.

**Methods:**

We retrospectively enrolled 39 trauma and postoperative patients who underwent Gd-EOB-DTPA-MRCP for suspected BL. Three readers rated the presence of BL and leakage site (intraparenchymal, central, peripheral ± aberrant or disconnected ducts). Imaging findings were compared to subsequent interventional procedures and their complexity and outcome.

**Results:**

BL was detected in Gd-EOB-DTPA-MRCP in 25 of patients and was subsequently confirmed. Sites of BL differed significantly between postoperative (central [58%] and peripheral [42%]) and trauma patients (intraparenchymal [100%]; *p* < 0.001). Aberrant or disconnected ducts were diagnosed in 8%/26% of cases in the postoperative subgroup. Inter-rater agreement for the detection and localization of BL was almost perfect (*Κ* = 0.85 and 0.88; *p* < 0.001). Intraparenchymal BL required significantly less complex interventional procedures (*p* = 0.002), whereas hospitalization and mortality did not differ between the subgroups (*p* > 0.05).

**Conclusions:**

Gd-EOB-DTPA-MRCP reliably detects and exactly locates BL in postoperative and trauma patients. Exact localization of biliary injuries enables specific treatment planning, as intraparenchymal leakages, which occur more frequently after trauma, require less complex interventions than central or peripheral leaks in the postoperative setting. As a result of specific treatment based on exact BL localization, there was no difference in the duration of hospitalization or mortality.

**Clinical relevance statement:**

Gd-EOB-DTPA-MRCP is a reliable diagnostic tool for exactly localizing iatrogenic and post-traumatic biliary leakage. Its precise localization helps tailor local therapies for different injury patterns, resulting in comparable clinical outcomes despite varying treatments.

**Key Points:**

*• Gd-EOB-DTPA-MRCP enables adequate detection and localization of bile leakages in both postoperative and post-traumatic patients.*

*• The site of bile leakage significantly impacts the complexity of required additional interventions.*

*• Intraparenchymal bile leakage is commonly seen in patients with a history of liver trauma and requires less complex interventions than postoperative central or peripheral bile leakages, while hospitalization and mortality are similar.*

## Introduction

Bile leakage (BL) following hepatobiliary surgery is a complication with growing importance due to a rising number of complex hepatic and biliary surgical procedures and can impact duration of hospitalization or cause liver failure [1, 2]. The most common non-iatrogenic cause of BL is abdominal trauma, resulting from penetrating (knife or gunshot wounds) or blunt injury with a wide range of reported incidences from 0.5 to 21% [3–6].

Initially, BL is often suspected when there is bile in abdominal drainages. Ultrasound, computed tomography (CT), and magnetic resonance imaging (MRI) with magnetic resonance cholangiopancreatography (MRCP) detect intra-abdominal fluid collections and assess their relationship to biliary structures. These primary imaging findings often trigger further investigations of the underlying cause, but are insufficient in providing evidence of active biliary leakage [7].

In routine clinical practice, the question of whether BL is present is often already answered by bile in the drains while various methods have been established to confirm and treat BL [6, 8]. However, for further clinical care, it is essential to know the site of leakage, which determines the treatment. Peripheral bile leaks can heal after percutaneous drainage of the biloma, whereas central leaks often require intraluminal drainage by stent or percutaneous transhepatic biliary drainage (PTBD). The situation is even more difficult with aberrant or disconnected bile ducts, as often neither biloma drainage nor bile duct stenting will help. Such patients can be treated locally by direct embolization or ablation and may ultimately require surgical revision.

Endoscopically, BL can be classified into low grade (identified only after intrahepatic opacification) or high grade (visible before intrahepatic opacification) [9]. Several classifications regarding anatomic site of bile duct injury, injury pattern, and the presence of aberrant ducts have been published [10]. On the other hand, so far, no radiological classification of BL which also considers treatment planning and clinical patient outcomes has yet been established.

With gadolinium ethoxybenzyl-diethylenetriaminepentaacetic (Gd-EOB-DTPA), approximately 50% of the contrast agent is taken up by hepatocytes and excreted by the biliary system [11]. Contrast-enhanced MRCP using a hepatocyte-specific contrast agent is a reliable non-invasive technique to confirm active BL and to evaluate biliary tree anatomy [12]. With Gd-EOB-DTPA-MRCP, the diagnosis of suspected BL is confirmed when contrast agent is visible in- and outside the biliary system on hepatobiliary phase imaging [13]. Biliary tree visualization is dependent on liver function and is achieved at approximately 20 min after intravenous Gd-EOB-DTPA administration in patients with normal liver function, and up to 60–180 min after administration in patients with impaired liver function [14, 15]. Today’s high spatial resolution of Gd-EOB-DTPA-MRCP should not only allow direct localization of the injured duct through the site of contrast extravasation but can also identify disconnected or aberrant bile ducts which could be missed on endoscopic retrograde cholangiopancreatography (ERCP) because they are not connected to the main biliary tree [16–18].

The purpose of this study was to assess the clinical relevance of Gd-EOB-DTPA-MRCP in the detection and exact localization of postoperative and post-traumatic BL. The two subgroups were compared retrospectively regarding the site of BL (intrahepatic, central and peripheral ± aberrant or disconnected ducts), duration of hospitalization, liver laboratory parameters, mortality, and complexity of required interventional procedures after imaging.

## Material and methods

### Patients

This retrospective study was approved by the regional committee for research ethics (Ethics Committee of Charité-Universitätsmedizin Berlin, EA 2/016/14). Our database search revealed 47 patients who were scheduled for Gd-EOB-DTPA-MRCP after hepatobiliary surgery or hospital admission due to abdominal trauma with liver injury between October 2016 and April 2021. Eight patients had to be excluded from the final analysis due to missing data after Gd-EOB-DTPA-MRCP. We thus included a total of 39 patients.

In postoperative patients, BL is clinically defined as fluid with an elevated bilirubin concentration in the abdominal drain (three times the serum bilirubin concentration measured at the same time) [19]. In the postoperative subgroup, BL was suspected according to this definition and clinical presentation, which included persistent fever more than 48 h after surgery, abdominal pain, bilious fluid from surgical drains, or altered liver function based on laboratory parameters. All patients in this subgroup underwent either ultrasound or diagnostic CT as primary imaging modalities, which showed perihepatic fluid collections suggestive of BL.

In the post-traumatic subgroup, patients presented with a history of abdominal trauma and underwent whole-body trauma CT using the local standard trauma protocol. BL was suspected when liver injury and adjacent fluid collections were visible on CT in conjunction with suggestive clinical findings or altered liver function on laboratory parameters. All patients in both subgroups then underwent Gd-EOB-DTPA-MRCP.

### Imaging

MRI was performed on a 1.5-Tesla scanner (MAGNETOM Aera, Siemens Healthineers) using a phased-array body coil with the patient in supine position using the following clinical imaging protocol: (1) in- and out-of-phase non-enhanced T1-weighted axial 3D gradient-echo (GRE) sequences; (2) coronal T2-weighted non-enhanced MRCP sequences; (3) non-enhanced fat-suppressed 3D GRE axial T1-weighted imaging; (4) intravenous administration of 9 mL Gd-EOB-DTPA (Primovist^®^, Bayer AG) followed by a 20-mL saline infusion at a rate of 2 mL/s through a peripheral venous catheter with a minimum size of 20G; (5) 3D GRE axial T1-weighted imaging with fat suppression 15, 50, 90, and 120 s after contrast administration; (6) axial T2-weighted fast spin-echo imaging; (7) 3D GRE axial T1-weighted imaging with fat suppression 4 min after contrast administration; (8) axial T2-weighted fast spin-echo sequence with fat suppression; (9) axial diffusion-weighted imaging; (10) sagittal T1-weighted fast low-angle shot imaging with a flip angle of 70°; (11) hepatobiliary phase imaging with a slice thickness of 3 mm or less: 3D GRE axial T1-weighted imaging with fat suppression 20 min (*n* = 20 patients), 30 min (*n* = 1 patients), or at both of these timepoints (*n* = 26 patients) after contrast administration; and (12) additional late-phase (> 240 min) 3D GRE axial T1-weighted images with fat suppression in patients with borderline biliary excretion and/or a dilated biliary tree (*n* = 7).

### Imaging analysis

Independent reading was performed by three radiologists with different levels of experience (A.A.M. 3 years; T.A.A. 6 years; U.F. 9 years) in abdominal MR imaging who were blinded to clinical data. Adequate biliary excretion was defined as visible contrast excretion into the biliary tree. BL was defined as contrast agent extravasation outside the biliary system during the hepatobiliary or late phase. BL was assigned to one of three sites: (1) intraparenchymal BL confined by surrounding liver tissue, (2) peripheral BL extending to or beyond the liver capsule including the hepatic resection margin, and (3) central BL due to injury of perihilar bile duct or the biliary confluence as well as leakage from a biliodigestive anastomosis. The presence of aberrant or postoperatively disconnected bile ducts was documented accordingly in a binary fashion (yes/no). In case of discrepancies between the readers after the initial blinded evaluation, a consensus reading was performed for further statistical evaluation. Findings were compared to subsequent ERCP, percutaneous transhepatic cholangiography (PTC), or surgical therapies.

Regarding the complexity of interventional procedures after MR imaging, we allocated patients with ERCP ± papillotomy or drainage change only to the “standard” group and patients with a history of ERCP and stent implantation, PTBD, radiofrequency ablation (RFA) of leakage site, biliary embolization, or percutaneous biloma drainage to the “complex” group.

Patient and laboratory data were retrieved from the local Radiology Information System (Centricity RIS-i, GE Healthcare).

### Statistics

Statistical analysis was performed using Stata/MP (Version 17.0, StataCorp). Patient characteristics are expressed as mean and range. Normal distribution was analyzed with the Kolmogorov-Smirnov test. The paired-sample *t*-test was performed to identify possible differences between two groups. The Wilcoxon rank-sum test was performed for non-parametric variables. Inter-rater variability was evaluated by kappa statistics. Statistical significance was defined as *p* ≤ 0.05.

## Results

Table 1 summarizes all anthropometric data and clinical parameters for the two subgroups. A total of 39 patients were enrolled (25 male and 14 female); seven were assigned to the post-traumatic and 32 patients to the postoperative subgroup. Mean age was 54.4 years for the total study population and differed significantly between the two subgroups with younger patients in the post-traumatic subgroup (*p* = 0.007). The most frequent initial indication for surgery in the postoperative subgroup was right hepatectomy in twelve patients, followed by atypical liver resection in seven patients and cholecystectomy in six patients. In the post-traumatic subgroup, five patients were admitted for blunt and two patients for penetrating liver trauma.

**Table 1:** Anthropometric data and clinical parameters for the trauma and postoperative subgroups, as well as for the total study population

Variable	Total study population (*n* = 39)	Trauma subgroup (*n* =7)	Postoperative subgroup (*n* = 32)	*p* value
Demographics (n) [%]				
Age (years) [min.-max.]	54.4 [7–81]	34.9 [15–53]	58.6 [7–81]	0.007
Gender				0.656
• Female	14 [36%]	2 [29%]	12 [37.5%]	
• Male	25 [64%]	5 [71%]	20 [72.5%]	
Diagnosis (n) [%]				
Blunt trauma		5 [71%]		
Penetrating trauma		2 [29%]		
Cholecystitis			6 [19%]	
Echinococcosis			1 [3%]	
Cirrhosis			1 [3%]	
Cholangiocellular carcinoma			8 [25%]	
Hepatocellular carcinoma			3 [9%]	
Gall bladder carcinoma			1 [3%]	
Liver metastasis			11 [34%]	
Hepatoblastoma			1 [3%]	
Surgical procedure (n) [%]				
Cholecystectomy			6 [19%]	
Right hepatectomy			12 [37.5%]	
Left hepatectomy			4 [12%]	
Atypical resection			7 [22%]	
Liver transplant			2 [6%]	
Clincal data (n) [%]				
Elevated liver parameters	8 [21%]	2 [29%]	6 [19%]	0.560
Bilious drain	16 [41%]	5 [71%]	11 [34%]	0.095
Fever	8 [21%]	3 [43%]	5 [16%]	0.106
Nausea	13 [33%]	3 [43%]	10 [31%]	0.555

**Fig. 1:**
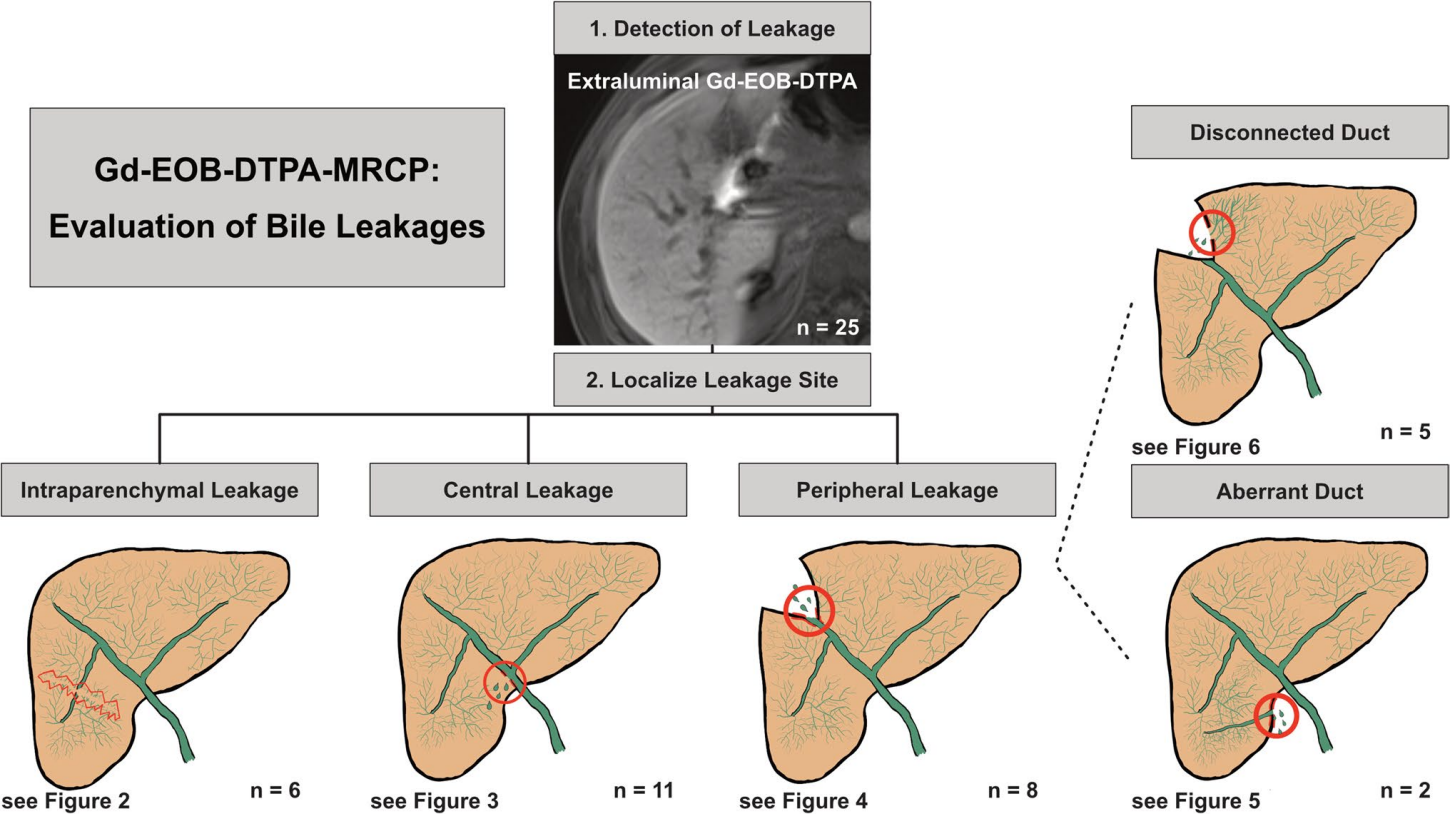
Illustrative overview of bile leakage evaluation by Gd-EOB-DTPA-MRCP

Results of retrospective Gd-EOB-DTPA-MRCP readings as well as data on subsequent interventions and outcome are summarized in Table 2. An illustrative overview for our diagnostic evaluation of BL with Gd-EOB-DTPA-MRCP is provided in Fig. 1. BL was identified in Gd-EOB-DTPA-MRCP in 25 patients (86% of patients in the post-traumatic and 59% of patients in the postoperative subgroup). Inter-rater agreement was almost perfect regarding presence (*Κ* = 0.85; *p* < 0.001) and localization of BL (*Κ* = 0.88; *p* < 0.001). Eighteen (72%) of the BL detected by Gd-EOB-DTPA-MRCP were interventionally confirmed by ERCP or PTC. When aberrant bile ducts or disconnected ducts are present, leakage cannot be detected by ERCP/PTC; in all of these cases (*n* = 7), ERCP/PTC ruled out leakage from ducts that were connected to the biliary tree. The diagnosis in these cases was also confirmed by persistent bile leakage via the indwelling drains. BL was visible in 100% of MRIs in the hepatobiliary phase (either 20 or 30 min after contrast agent administration) in patients with adequate biliary excretion. Seven patients had inadequate hepatic contrast agent excretion in hepatobiliary phase imaging after 30 min (three patients with poor or borderline biliary excretion and four patients with dilated bile ducts). Three of these seven patients showed BL detected exclusively in very late-phase images (> 240 min). Mean time from hospital admission (post-traumatic subgroup) or surgery (postoperative subgroup) to Gd-EOB-DTPA-MRCP was 17.6 days. In the trauma subgroup, Gd-EOB-DTPA-MRCP was performed significantly earlier (7.3days vs. 19.9days; *p* = 0.040). Elevated liver function parameters (either liver enzymes or bilirubin) were detected in eight patients prior to the MRI examination (< 48 h). There was no significant correlation between elevated liver laboratory parameters and the presence of BL (*p* = 0.916). Sites of BL differed significantly between the two subgroups: all post-traumatic patients were diagnosed with intraparenchymal BL (100%), while central (58% of postoperative subgroup) and peripheral (42% of postoperative subgroup) BL were observed in postoperative patients only (*p* < 0.001). In five patients of our cohort with confirmed peripheral BL, Gd-EOB-DTPA-MRCP detected the presence of leakage from disconnected bile ducts after surgery. Three of these patients were successfully treated with RFA, the other two patients underwent a percutaneous biliary embolization, which led to subsidence of the perihepatic biloma. An aberrant bile duct responsible for the leakage was identified in two patients, treated by percutaneous embolization in one case and percutaneous biloma drainage in the other.

**Table 2:** Summary of Gd-EOB-DTPA-MRCP findings and data on treatment/outcome

Variable	Total study population (*n* = 39)	Trauma subgroup (*n* = 7)	Postoperative subgroup (*n* = 32)	*p* value
Gd-EOB-DTPA-MRCP (n) [%]				
Time to Gd-EOB-DTPA-MRCP (d) [min.-max.]	17.6 [1–63]	7.3 [1–22]	19.9 [1–63]	0.040
Bile Leak in Gd-EOB-DTPA-MRCP	25 [64%]	6 [86%]	19 [59%]	0.188
Site of bile leak				< 0.001
• Intraparenchymal	6 [24%]	6 [100%]	0	
• Central	11 [44%]	0	11 [58%]	
• Peripheral	8 [32%]	0	8 [42%]	
Aberrant duct	2 [8%]	0	2 [11%]	
Disconnected duct	5 [20%]	0	5 [26%]	
Treatment (n) [%]				
No Intervention	7 [28%]	2 [33%]	5 [26%]	0.739
Standard Procedures	4 [16%]	4 [66%]	0	0.002
• ERCP ± papillotomy	2 [50%]	2 [50%]	0	
• Drain Change	2 [50%]	2 [50%]	0	
Complex procedures	14 [56%]	0	14 [74%]	
• Biliary stent	6 [43%]	0	6 [43%]	
• Ablation	3 [21%]	0	3 [21%]	
• PTBD	2 [14%]	0	2 [14%]	
• Percutaneous drainage	2 [14%]	0	2 [14%]	
• Embolization	2 [14%]	0	3 [21%]	
• Biliodigestive Anastomosis	1 [7%]	0	1 [7%]	
Hospitalization (d) [min.-max.]	34.8 [8–92]	28.8 [8–48]	36.6 [10–92]	0.390
30-day mortality (*n*) [%]	2 [8%]	0	2 [11%]	0.407

*Gd-EOB-DTPA* gadolinium ethoxybenzyl-diethylenetriaminepentaacetic acid, *ERCP* endoscopic retrograde cholangioancreatography, *PTBD* percutaneous transhepatic biliary drainage

Patients in the postoperative subgroup underwent significantly more complex interventional procedures after Gd-EOB-DTPA-MRCP-based diagnosis of BL compared to trauma patients (*p* = 0.002). Of six patients with confirmed BL in the trauma subgroup, two patients did not need further interventions, two patients underwent ERCP and papillotomy and another two patients received a simple change of drainages (inserted during emergency surgery performed before MRI). No patient with traumatic intraparenchymal BL required complex local treatment. Conversely, 14 of 19 patients (74%) with confirmed BL in the postoperative subgroup underwent complex interventional procedures including biliary stenting (43%), ablation of disconnected biliary ducts (21%), PTBD (14%), percutaneous biloma drainage (14%), biliary embolization of aberrant/disconnected ducts (14%), and one patient needed surgical revision due to the size and central location of the biliary defect. There were no significant differences in duration of hospitalization and mortality between the two subgroups (*p* = 0.390).

## Discussion

In this retrospective single-center study, we investigated the clinical impact of Gd-EOB-DTPA-MRCP in the exact localization of BL in patients after hepatobiliary surgery or liver trauma. The main findings were (1) Gd-EOB-DTPA-MRCP enables the detection and precise localization of BL, in both postoperative and trauma patients; (2) Gd-EOB-DTPA-MRCP-based detection and localization show high agreement between readers with different levels of experience; (3) intraparenchymal BL is typical for a history of liver trauma whereas central or peripheral BL is more common after hepatobiliary surgery; (4) patients with intraparenchymal BL require fewer complex interventional procedures than patients with central or peripheral BL following hepatobiliary surgery; (5) although treatment differs significantly for different sites of BL, Gd-EOB-DTPA-MRCP allows adequate treatment planning, so that there are no significant differences in hospitalization duration and mortality.

**Fig. 2:**
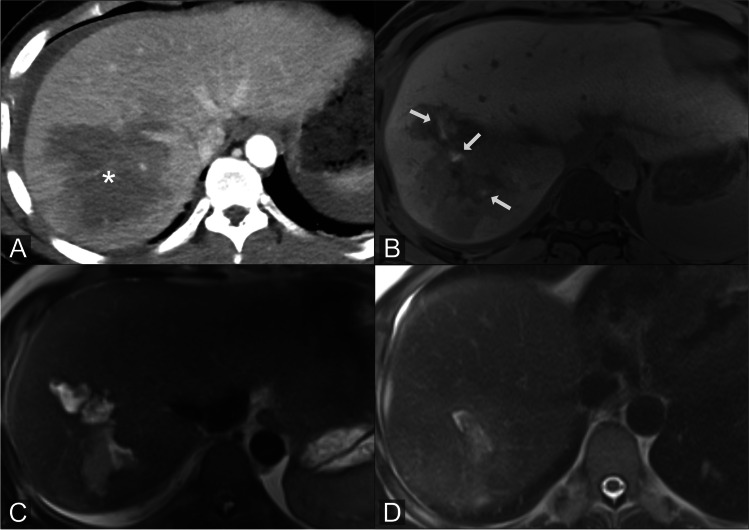
Intraparenchymal bile leakage: 32-year-old patient admitted to our hospital with blunt liver trauma. Initial CT scan shows laceration of the right liver lobe (**A**). Gd-EOB-DTPA-MRCP performed 4 days later shows intraparenchymal BL with Gd-EOB-DTPA extravasations (arrows) (**B**). T2w image shows the extent of the parenchymal lazeration (**C**). MRI follow-up 5 weeks after initial trauma: T2w image shows significant decrease of intrahepatic lazeration (**D**). No further intervention was required

In our study, Gd-EOB-DTPA-MRCP detected all cases of ultimately proven BL in hepatobiliary and late-phase imaging and accurately identified leakage sites in a relatively large population. Similar findings were reported by Castellanos et al, who detected 100% of bile leaks in postoperative patients by Gd-EOB-DTPA-MRCP 20 min after contrast agent administration [12]. Moreover, our study confirmed results of Cieszanowski et al, who observed that additional late-phase Gd-EOB-DTPA-MRCP (60–180 min) identifies BL in patients with inadequate biliary excretion due to impaired liver function [14]. Our study underlines the value of Gd-EOB-DTPA-MRCP for non-invasive confirmation of biliary leakage and supports its robustness in clinical routine by showing an almost perfect inter-rater agreement [12, 17, 18, 20, 21]. In our cohort, liver laboratory parameters were not indicative of the presence or absence of a BL, which further corroborates the value of Gd-EOB-DTPA-MRCP. Apart from the established benefit of Gd-EOB-DTPA-MRCP in postoperative BL, our study also highlights its diagnostic accuracy in trauma patients. Our results show that the biliary injury pattern of trauma patients differs significantly from that of postoperative bile leakage. Bile leakage after trauma is typically located within the parenchyma. Coverage by surrounding liver parenchyma prevents leakage of bile into the peritoneum, and thus the risk of biliary peritonitis or formation of large bilomas should be lower than in patients with injuries to biliary structures not surrounded by liver tissue [22] (Fig. 2). Our results indirectly prove this assumption, as the treatment of intraparenchymal BL required significantly less complex interventions than peripheral or central bile duct injuries. And our results also suggest that intraparenchymal BL has the potential to resolve without further complex surgical or radiological intervention. Of course, factors such as age, co-morbidities, and size of liver laceration might influence these findings.

**Fig. 3:**
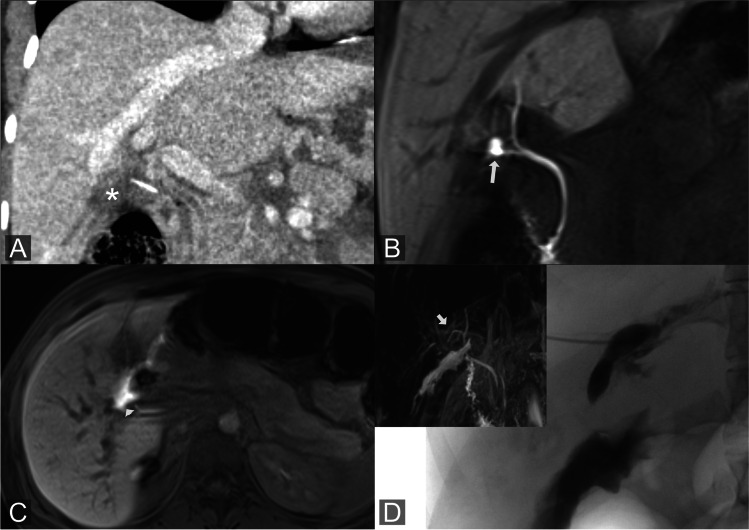
Central bile leakage: CT of a 42-year-old patient with a small subhepatic postoperative biloma (asterisk) after laparoscopic cholecystectomy (**A**). Gd-EOB-DTPA-MRCP in coronal plane shows extraluminal Gd-EOB-DTPA at the central right hepatic duct (arrow) with suspected injury (**B**). Axial plane depicts the extensive Gd-EOB-DTPA extravasation at the liver hilum (arrow head) (**C**). After unsuccessful ERCP, percutaneous cholangiogram of a right segmental duct confirms the high volume, central leakage of the right main hepatic duct (left corner: T2w-MRCP shows the anatomy of the whole biliary tree; arrow: entry point of the PTC canula). The extrahepatic common hepatic duct could not be cannulated across the large defect, so that no sufficient drainage could be achieved. Ultimately, this patient had to undergo surgical revision with creation of a biliodigestive anastomosis (**D**)

**Fig. 4:**
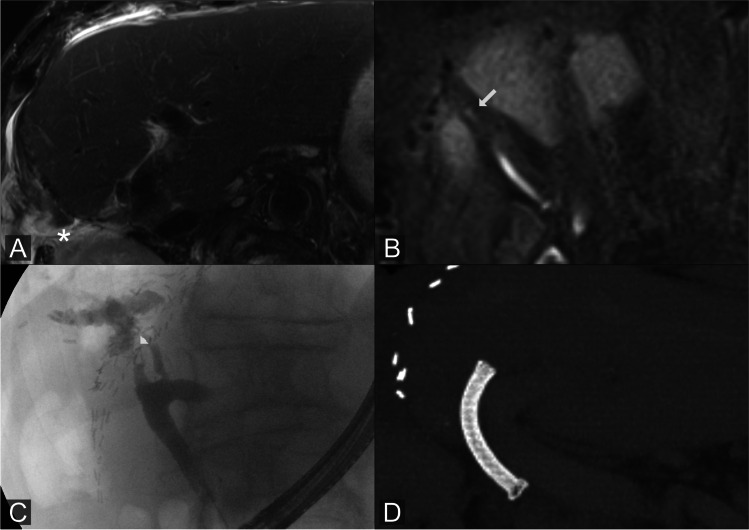
Peripheral bile leakage: MRI of a 70-year-old patient with colorectal liver metastasis after right hemihepatectomy. T2w image shows diffuse perihepatic fluid (asterisk) without formation of a biloma (**A**). Gd-EOB-DTPA-MRCP reveals a small extraluminal contrast agent collection at the surgical margin originating from the right hepatic duct (**B**). ERCP confirms the peripheral leakage at the resection margin (**C**). The patient was treated by endoscopic biliary stenting; the intraluminal metal stent covering the leakage site is visualized in this post-interventional CT maximum intensity projection (MIP) (**D**)

On the other hand, central and peripheral BL is more frequently seen in postoperative patients, who required more complex interventions than intraparenchymal BL. This could be attributable to the unconstrained excretion of bile into the adjacent peritoneal cavity with a higher risk of biliary peritonitis. Central and peripheral leaks are typically treated with endoscopic stents or, in the case of biliodigestive anastomosis, with PTBD. In rare cases, however, the bile duct defect is so large that surgical revision is necessary (Fig. 3). Peripheral leakages often occur at the resection margin and, like central leaks, are often treated endoscopically or percutaneously with stents/drains (Fig. 4). Percutaneous biloma drainage is often also necessary here. In our study population, 93% of postoperative bile leaks could be treated percutaneously or endoscopically.

**Fig. 5:**
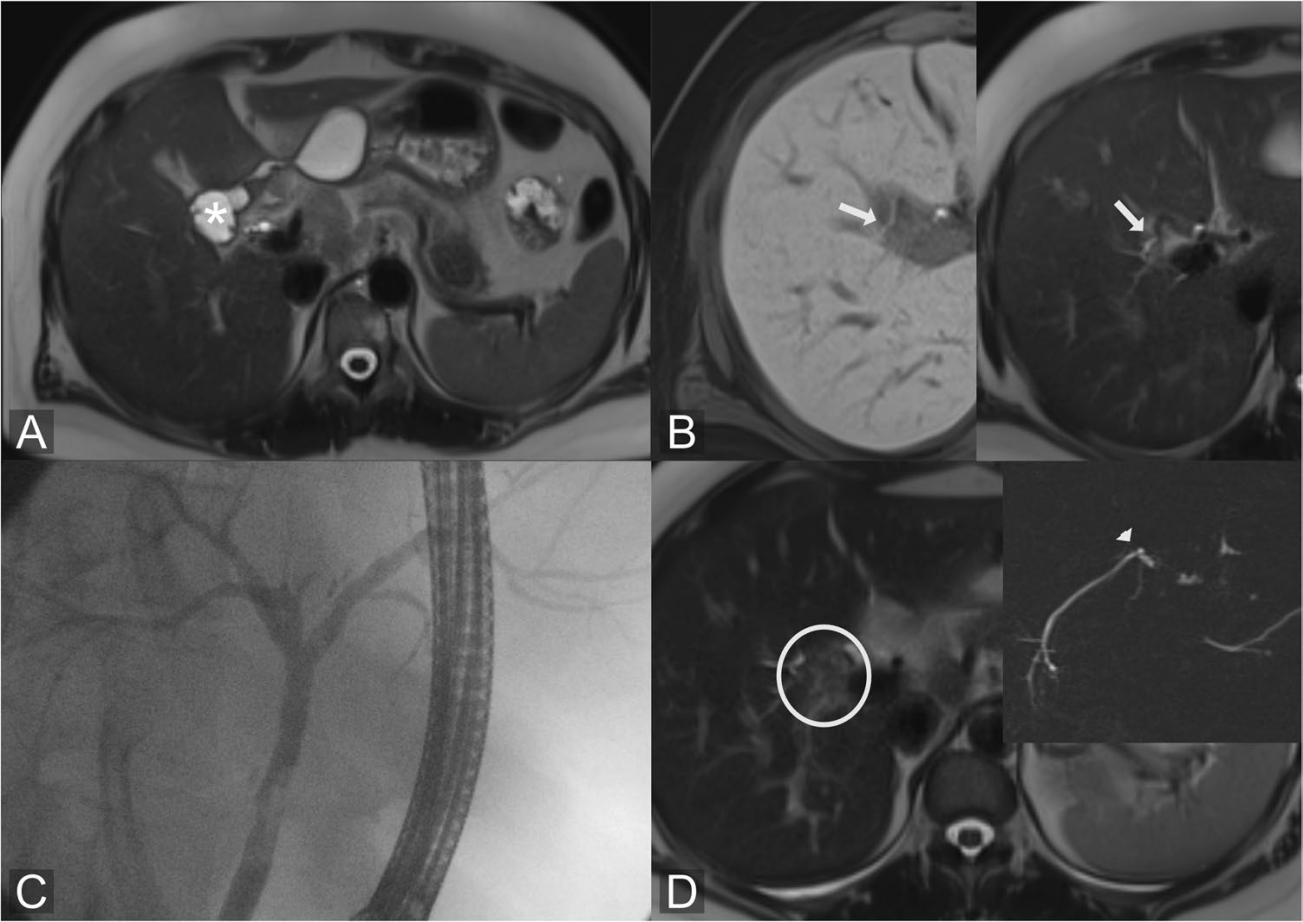
Aberrant duct: MRI of a 39-year-old patient after cholecystectomy. T2w image shows a biloma (asterisk) in the gall bladder fossa (**A**). Gd-EOB-DTPA-MRCP reveals contrast extravasation into the gallbladder fossa originating from an aberrant duct in liver segment 5 (arrow) which is also visible in T2w image (**B**). ERCP shows normal findings (**C**). Patient was treated by percutaneous biloma drainage. MRI 5 weeks after therapy shows complete regression of the biloma and post-inflammatory scar tissue at the former leakage site (circle), resulting in non-symptomatic dilatation of the corresponding aberrant bile duct in MRCP (arrowhead) (**D**)

**Fig. 6:**
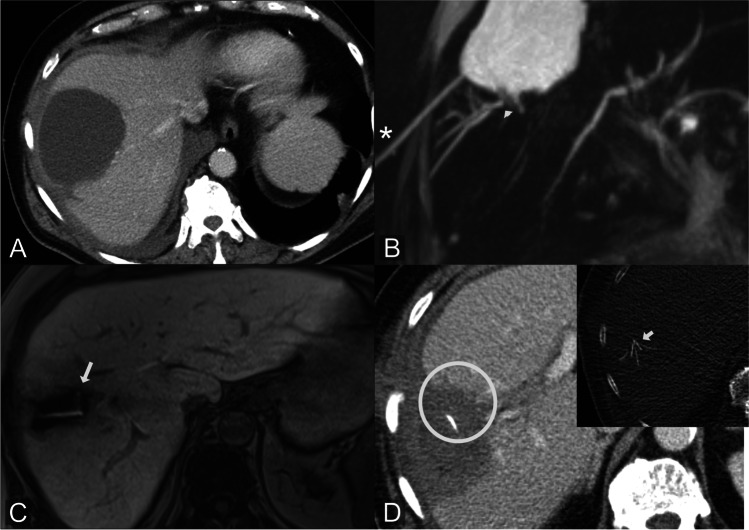
Disconnected duct: CT of a 71-year-old patient with colorectal liver metastases after surgical enucleation and postoperative biloma (**A**). T2w-MRCP demonstrates the biloma after percutaneous drainage (asterisk) and displays a disconnected segmental bile duct opening into the biloma (arrowhead) (**B**). Gd-EOB-DTPA-MRCP proves peripheral BL from this disconnected duct into the biloma at the resection site (**C**). Leakage was subsequently treated by radiofrequency ablation of the disconnected duct. CT fluoroscopy of probe placement (short arrow) and the corresponding ablation zone in post-interventional contrast-enhanced CT are shown (**D**)

A major challenge in hepatobiliary surgery, both diagnostically and therapeutically, is leakage from disconnected or aberrant bile ducts as these leaks usually cannot be treated by an endoscopically placed stent or PTBD. In our study, Gd-EOB-DTPA-MRCP could identified such ducts in seven patients with peripheral BL. In these cases, imaging findings led to the decision to perform RFA/embolization instead of standard internal or external drainage [23] (Figs. 5 and 6).

Our study has some limitations. The two groups we investigated differed significantly in age and preexisting conditions, which might have confounded the results, specifically clinical outcome. Interventional confirmation of the imaging diagnosis was only evaluated when BL was suspected in Gd-EOB-DTPA-MRCP, so that the sensitivity of the method could not be determined, nor was this our intention as several studies on this topic have already confirmed the high sensitivity of Gd-EOB-DTPA-MRCP [24]. Moreover, the total number of patients in general and the small number of patients with post-traumatic BL in particular as well as the retrospective and single center study design limit the generalizability of our findings.

In conclusion, Gd-EOB-DTPA-MRCP is a reliable noninvasive method to detect and to exactly localize iatrogenic and post-traumatic BL, which typically differ in the site of biliary injury. Intraparenchymal leakages, which occur more frequently after trauma, require less complex interventions compared to central or peripheral postoperative BL. The exact localization of the leakage by Gd-EOB-DTPA-MRCP makes it possible to plan a tailored local therapy for specific injuries, so that comparable clinical outcomes can be achieved despite different treatments.

## Abbreviations

BL: Bile leakage
ERCP: Endoscopic retrograde cholangiopancreatography
Gd-EOB-DTPA: Gadolinium ethoxybenzyl-diethylenetriaminepentaacetic acid
PTBD: Percutaneous transhepatic biliary drainage
PTC: Percutaneous transhepatic cholangiopancreatography
RFA: Radiofrequency ablation

## Declarations

### Guarantor

The scientific guarantor of this publication is Uli Fehrenbach, MD.

### Statistics and biometry

One of the authors has significant statistical expertise.

### Study subjects or cohorts overlap:

No study subjects or cohorts have been previously reported.

### Methodology

- retrospective
- observational study
- performed at one institution
